# Predicting the incidence of mild cognitive impairment with a computer-based cognitive assessment tool in community-dwelling older adults: The Otassha study

**DOI:** 10.1371/journal.pone.0297433

**Published:** 2024-01-25

**Authors:** Junta Takahashi, Hisashi Kawai, Manami Ejiri, Yoshinori Fujiwara, Hirohiko Hirano, Hiroyuki Sasai, Shuichi Obuchi

**Affiliations:** 1 Exercise Motivation and Physical Function Augmentation Research Team, National Institute of Advanced Industrial Science and Technology, Tokyo, Japan; 2 Tokyo Metropolitan Institute of Gerontology, Tokyo, Japan; University of Bari Department of Education Psychology and Communications: Universita degli Studi di Bari Aldo Moro Dipartimento di Scienze della Formazione Psicologia Comunicazione, ITALY

## Abstract

This study examined the ability of a computer-based cognitive assessment tool (CompBased-CAT) to predict mild cognitive impairment (MCI) in community-dwelling older adults. A two-year longitudinal study was conducted using data from 2016 to 2018 from the Otassha study cohort of community-dwelling older adults. MCI was defined as a Mini-Mental Status Examination (MMSE) score of <27. The CompBased-CAT was used at baseline, with each subtest score converted into a Z-score. Subsequently, the total Z-scores were calculated. Participants were divided into robust and MCI groups, and all variables were compared using the *t*-test or χ^2^ test. Receiver operating characteristic (ROC) curves and logistic regression analyses were conducted, with MCI and total Z-scores as dependent and independent variables, respectively. Among the 455 participants (median age, 72 years; range, 65–89 years; 282 women and 173 men), 32 developed MCI after two years. The participants in the MCI group were significantly older. They had lower maximal gait speed, baseline MMSE scores, subtest Z-scores, and total Z-scores than those in the robust group. The area under the ROC curve was 0.79 (95% confidence interval: 0.70–0.87; *P* <0.01). The sensitivity was 0.76, and the specificity was 0.75. The logistic regression analysis showed an odds ratio of 1.34 (95% confidence interval: 1.18–1.52; *P* <0.01). This study showed that CompBased-CAT can detect MCI, which is an early stage of dementia. Thus, CompBased-CAT can be used in future community health checkups and events for older adults.

## Introduction

As the global population continues to age, the number of people with dementia has been increasing, with approximately 1.52 billion people estimated to have dementia by 2050 [1]. The decline in the quality of life (QOL) of patients with dementia, the burden of care assumed by their families, and socioeconomic burden have emerged as major concerns [2, 3], making dementia prevention an urgent issue.

Approximately 10–15% of individuals with mild cognitive impairment (MCI) progress to Alzheimer’s disease annually [4]; therefore, early diagnosis and management of MCI is important in preventing dementia. Approximately 35% of risk factors for dementia are modifiable [5], and interventions such as a Mediterranean diet [6], exercise [7], and social participation [8] have been reported to reduce the risk of dementia. Therefore, the early detection of MCI can be expected to reduce the risk of developing dementia or delay its progression with appropriate interventions and social support. Earlier interventions provided people with dementia and their families with more time to prepare for the future [5]. However, dementia is often not diagnosed until its late stages, rendering the diagnosis considerably less useful [9]. Delays of several years in screening and diagnosis can reduce the effectiveness of interventions. Therefore, cognitive impairments should be detected as early as possible. Currently, paper-based tests such as the Mini-Mental State Examination (MMSE) [10] and Montreal Cognitive Assessment (MoCA) [11] are widely used to screen for MCI. However, paper-based assessment tools have disadvantages such as learning effectiveness, measurement bias, and the requirement of trained staff [12], making them unsuitable for frequently testing older adults or validating the effectiveness of interventions.

Therefore, we developed a computer-based cognitive assessment tool (CompBased-CAT), a cognitive function testing tool that uses a tablet computer [13]. The tool does not require a trained examiner and has no measurement bias because the examinee is guided and assessed using a computer. In addition, the tasks were selected randomly; therefore, the learning effect was considered. Our previous study [13] revealed that this tool moderately correlated with the MMSE score (***r*** = 0.51) and had sufficient discriminating ability for dementia (sensitivity = 0.81, specificity = 0.77). However, the previous study was cross-sectional, and whether the CompBased-CAT can predict future MCI remains unclear. Although previous studies have used tablet computers to identify the occurrence of dementia with advanced cognitive impairment, no study has examined MCI. Detecting risk at the MCI stage is potentially useful for dementia prevention.

This study aimed to examine the ability of the CompBased-CAT to predict the future occurrence of MCI in community-dwelling older adults. Moreover, the association between CompBased-CAT scores at baseline and the occurrence of MCI two years later was examined. We hypothesize that CompBased-CAT has a practical predictive ability for future MCI incidence.

## Materials and methods

### Study design and participants

We conducted a two-year longitudinal study. Participants were selected from the 2016 survey of the Otassha Study [14]. This cohort study began in 2011 and is conducted annually in September and October at the Tokyo Metropolitan Institute of Gerontology (Tokyo, Japan). Participants were recruited by sending invitations to community-dwelling older adults aged ≥65 years living in nine areas of Itabashi Ward, Tokyo. Invitations were sent annually to those who had previously participated in this study and those who were newly 65 (except in 2015). Participants were recruited for the 2016 survey between October 19 and November 10, 2016. The inclusion criteria were participation in the 2016 and 2018 surveys and the absence of MCI at baseline (MMSE score ≥27). In contrast, those with a history of dementia and those with missing data were excluded. The presence of dementia was verified through an interview with a nurse, checking for medical history and medications. The participants were informed of the study in advance, and their written informed consent was subsequently obtained. We did not have access to information that could identify the individual participants during or after data collection. The study protocol was approved by the Ethics Committee of the Tokyo Metropolitan Institute of Gerontology (approval number 2015–18).

### CompBased-CAT measurement

The CompBased-CAT is a multidomain cognitive function test that uses a tablet computer (ASUS TransBook T100HA China, OS: Windows 10) and consists of six subtests: digit span forward (attention and concentration), digit span backward (attention and concentration), memory of item names (immediate memory), memory recall of item names (remote memory), Stroop (executive function and selective attention), and recognition of figures (space perception) [13]. The participants wore noise-canceling headphones and responded to the tasks while receiving audio guidance. They first performed practice tasks and subsequently performed the main task. The CompBased-CAT was conducted at an inspection venue during the 2016 survey. The CompBased-CAT scores were converted to Z-scores for each subtest based on data from the overall study population, and the total Z-score was calculated by adding the Z-scores of all subtests without any weighting [13].

### Measuring the occurrence of cognitive impairment

Cognitive function was evaluated using the MMSE. MCI was defined as an MMSE score of <27. The discriminatory ability of the MMSE for MCI when using this cut-off value has been reported previously, with sensitivities and specificities of 69.1–85.7% and 77.3–80.1%, respectively [15]. The MMSE was administered through face-to-face interviews with trained staff at inspection venues in 2016 and 2018.

### Other measurements

Covariates including age, sex, number of chronic diseases (cerebral stroke, cardiac diseases, diabetes mellitus, and depression), years of education, maximal gait speed, simple Japanese version of the World Health Organization (WHO)-Five well-being index (WHO-5) [16], and Tokyo Metropolitan Institute of Gerontology Index of Competence (TMIG-IC) [17] at the time of the 2016 survey were assessed. The WHO-5 is a short version of the WHO Well-Being Scale that was initially developed to evaluate the quality of care for diabetes and has been validated in the context of various health states, including depression, anxiety, psychiatric disorders, and health-related QOL. Scores range from 0 to 15, with higher scores indicating better mental health [16]. The TMIG-IC was designed based on Lawton’s model of human behavior and was used to assess the ability of older adults to perform instrumental activities of daily living (IADL). The scores range from 0 to 13, with higher scores indicating better IADL ability [17]. Maximum gait speed was calculated by measuring the time required to walk a 5 m path with maximum effort. Preliminary 3 m paths for acceleration and deceleration were provided at the beginning and end. Walking speed was measured twice, and the maximum value was adopted. The other items were acquired through face-to-face interviews with the researcher.

### Statistical analysis

Participants were divided into robust and MCI groups, and all variables were compared between the groups using the *t*-test or χ^2^ test. Receiver operating characteristic (ROC) curve analysis was performed to predict the occurrence of MCI after two years based on the total Z-score of the CompBased-CAT, and the area under the curve (AUC) was calculated. The cut-off point, sensitivity, and specificity were calculated using the Youden index. As a sub-analysis, logistic regression analysis was performed to determine the association between CompBased-CAT scores (total Z-scores and subtest scores) and the occurrence of MCI. In model 1, age, sex, and years of education were included as covariates. In model 2, the number of chronic diseases (cerebral stroke, cardiac diseases, diabetes mellitus, and depression), grip strength, maximum gait speed, WHO-5 score, and TMIG-IC score were included as additional covariates. Statistical significance was set at *P* <0.05. All analyses were performed using the IBM SPSS software (version 25.0; IBM Japan, Tokyo, Japan).

## Results

### Participant characteristics

**Fig 1:**
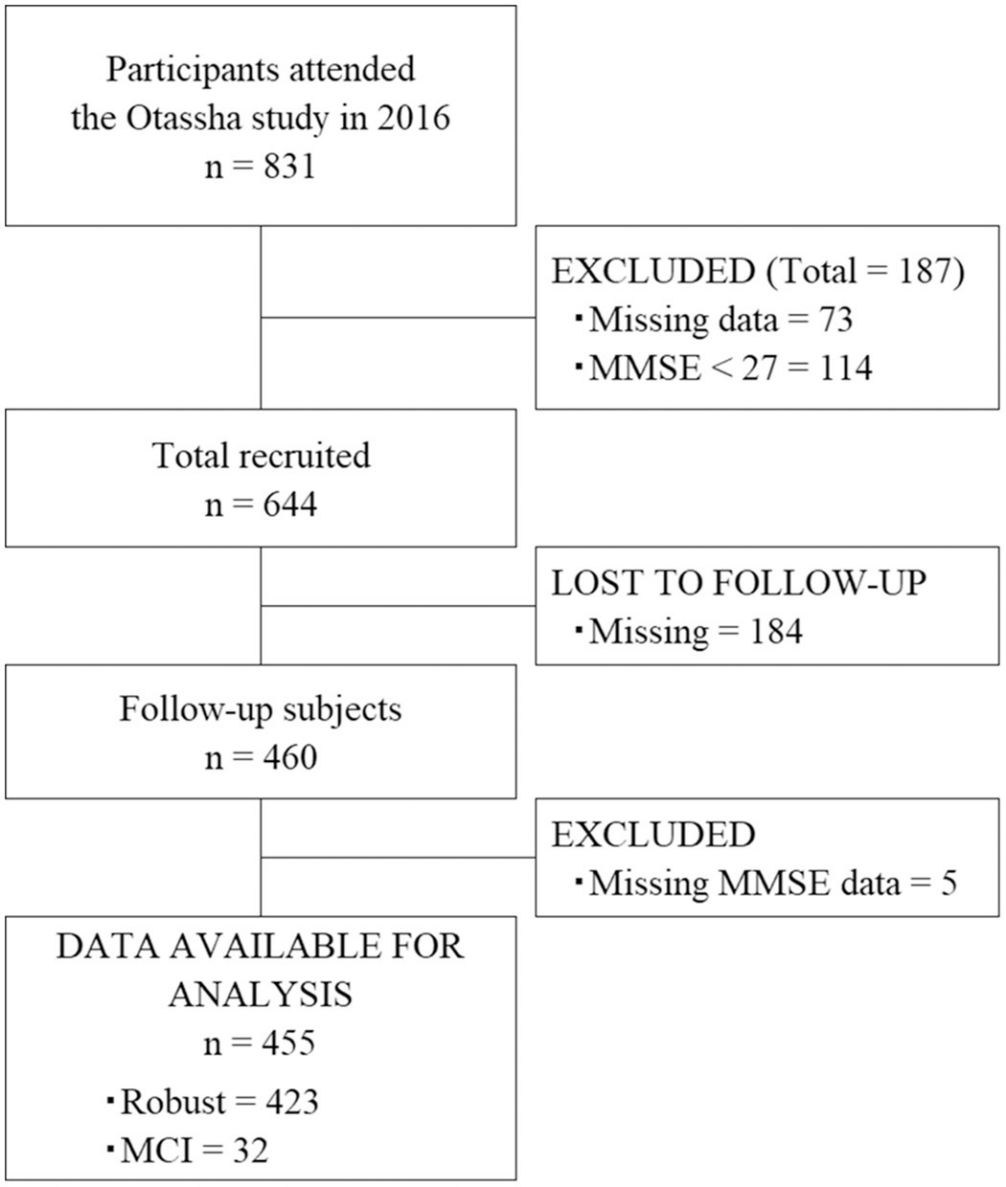
Flowchart of participants.

In the baseline survey, the total number of participants were 831, of whom 187 were excluded (missing data: 73; MMSE score: <27:114). Of the 644 eligible participants at baseline, 184 (28.5%) were lost to follow-up. Finally, 455 participants were included in the analysis (median age: 72 years; range: 65–89 years; 282 women and 173 men), 32 of whom had new MCI (Fig 1, Table 1). The mean years of education for all participants were 13.4 ± 2.8 years. The mean MMSE was 29.0 ± 1.0, and the mean score for TMIG-IC was 12.5 ± 1.0. Participants in the MCI group were significantly older and had lower maximum gait speed, baseline MMSE scores, subtest Z-scores, and total Z-scores than those in the control group. Table 2 shows the CompBased-CAT scores at baseline. The total Z-score ranged from −13.7 to 7.42, and the mean total Z-score was 0.27 ± 3.26. The Z-scores in all subtests and the total Z-score from the CompBased-CAT were significantly lower in the MCI group than in the robust group.

**Table 1:** Characteristics of the robust and MCI groups at baseline (2016 survey).

Variables	Values			*P*-value
	All (n = 455)	Robust (n = 423)	MCI (n = 32)	
**Median age, years (range)**	72 (65–89)	71 (65–89)	75.5 (66–88)	<0.01
**Women, n (%)**	282 (62)	260 (61.5)	22 (68.8)	0.41
**Education, years (SD)**	13.4 (2.8)	13.5 (2.8)	12.5 (2.5)	0.06
**Chronic disease, n (%)**				
Cerebral stroke	25 (6)	23 (5)	2 (6)	0.85
Cardiac diseases	55 (12)	53 (13)	2 (6)	0.29
Diabetes mellitus	50 (11)	45 (11)	5 (16)	0.38
Depression	21 (5)	19 (5)	2 (6)	0.65
**Grip strength, kgf (SD)**	27.0 (8.5)	27.1 (8.5)	24.9 (9.1)	0.17
**Maximum gait speed, m/s (SD)**	2.15 (0.39)	2.16 (0.38)	1.91 (0.40)	<0.01
**WHO-5 (SD)**	16.6 (4.7)	16.7 (4.5)	15.8 (6.1)	.40
**MMSE (SD)**	29.0 (1.0)	29.0 (1.0)	28.2 (1.1)	<0.01
**TMIG-IC (SD)**	12.5 (1.0)	12.5 (1.0)	12.3 (1.1)	0.27

WHO-5, Simplified Japanese version of the WHO-Five well-being index; MMSE, Mini-Mental State Examination; TMIG-IC, Tokyo Metropolitan Institute of Gerontology Index of Competence; SD, standard deviation

**Table 2:** Comparison of CompBased-CAT scores between the robust and MCI groups at baseline (2016 survey).

Variables	Score range	Robust				MCI				*P*-value
		Mean	SD	Min	Max	Mean	SD	Min	Max	
**Digit span forward**	0–8	5.6	1.3	3	8	4.4	1.4	3	8	<0.01
**Digit span backward**	0–8	4.7	1.4	0	8	3.5	1.3	0	6	<0.01
**Memory of the item name**	0–10	9.1	0.9	5	10	8.6	1.2	5	10	0.03
**Memory recall of the item name**	0–10	7.7	3.0	0	10	5.6	4.2	0	10	0.01
**Stroop task**	0–20	16.7	5.6	1	20	13.8	6.8	3	20	0.02
**Recognition of figures**	0–6	4.9	1.5	0	6	4.1	2.0	0	6	0.02
**Total Z-score**	N/A	0.27	3.26	-12.3	7.42	-3.62	3.80	-13.7	3.49	<0.01

SD; standard deviation; MCI, mild cognitive impairment

### Predictability of the CompBased-CAT

**Fig 2:**
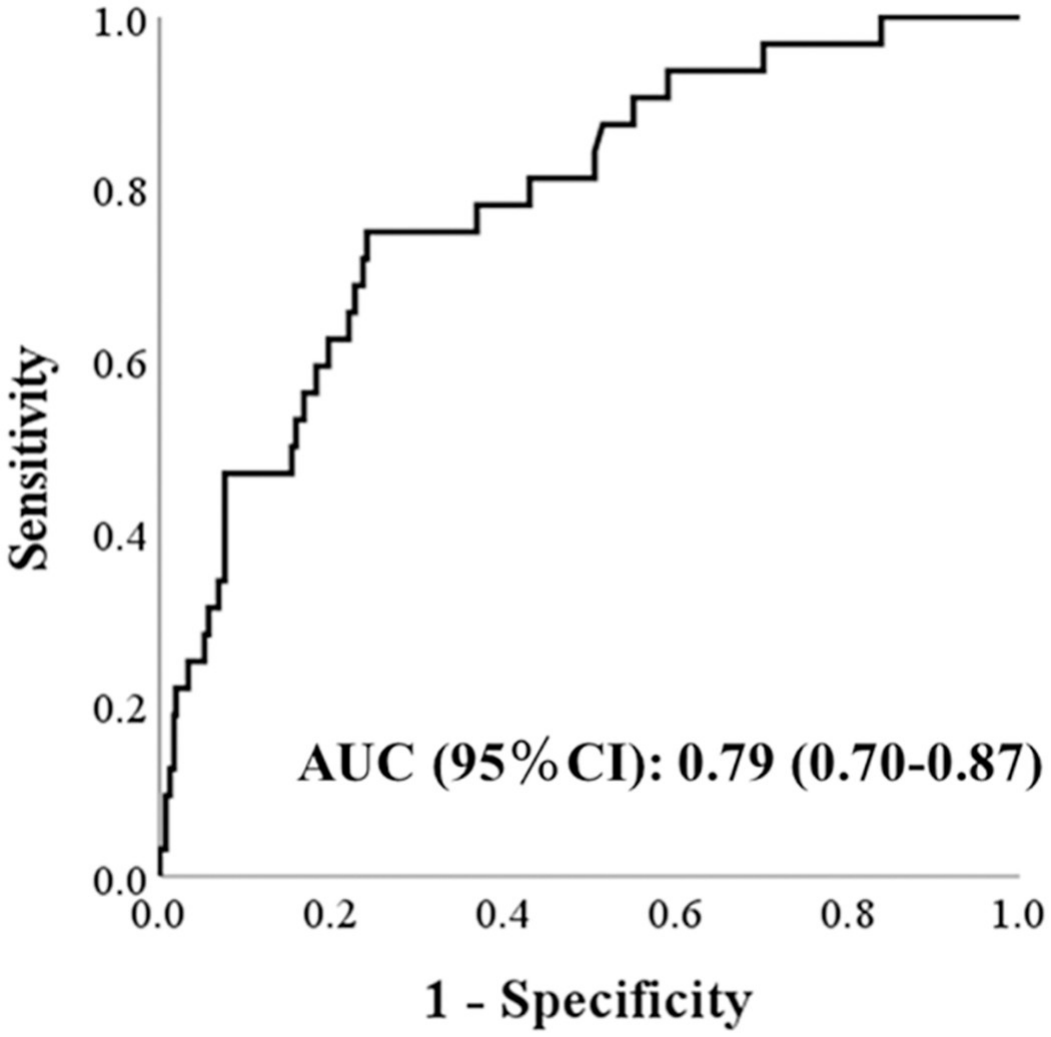
ROC curve for predicting the occurrence of MCI (MMSE <27) within two years after assessment, based on total Z-scores from the CompBased-CAT. AUC, area under the receiver operating characteristic curve; CI, confidence interval.

Fig 2 shows the ROC curves for predicting the occurrence of MCI and the performance indicators of CompBased-CAT. The AUC was 0.79 (95%CI: 0.70–0.87, *P* <0.01), and the cut-off total Z score was -1.67, with sensitivity and specificity of 0.76 and 0.75, respectively.

### Association between CompBased-CAT scores and the risk of MCI

The total and subtest Z-scores of the CompBased-CAT were significantly associated with MCI risk in the crude model and model 1 (Table 3). In model 2, significant associations were observed for the total Z-score, digit span forward, digit span backward, memory recall of item names, recognition of figures, and Stroop task. However, no significant associations were observed for the memory of item names. In comparisons by subtest, odds ratios were higher for the digit span backward, digit span forward, memory recall of an item name, recognition of figures, and the Stroop task.

**Table 3:** Logistic regression analysis of the association between the CompBased-CAT and occurrence of MCI within two years.

Variables	Crude model			Model 1			Model 2		
	OR	95% CI	***P***-value	OR	95% CI	***P***-value	OR	95% CI	***P***-value
**Total Z-score**	1.36	1.22–1.52	< .01	1.36	1.20–1.53	< .01	1.34	1.18–1.52	< .01
**Subtests**									
Digit span forward	2.34	1.60–3.42	< .01	2.12	1.40–3.20	< .01	2.11	1.38–3.23	< .01
Digit span backward	2.70	1.70–4.28	< .01	2.45	1.51–3.98	< .01	2.21	1.35–3.63	< .01
Memory of the item name	1.55	1.13–2.12	< .01	1.43	1.03–1.98	.03	1.30	0.91–1.84	.15
Memory recall of the item name	1.66	1.25–2.20	< .01	1.56	1.15–2.11	< .01	1.54	1.12–2.10	< .01
Stroop task	1.52	1.13–2.06	< .01	1.42	1.03–1.94	.03	1.41	1.02–1.94	.04
Recognition of figures	1.53	1.16–2.03	< .01	1.50	1.12–2.01	< .01	1.49	1.10–2.01	.01

Independent variables: total Z-scores and Z-scores for each CompBased-CAT subscale

Dependent variable: occurrence of MCI at two years (reference: MCI occurrence)

Covariates: model 1: age, sex, and education; model 2: number of chronic diseases, grip strength, maximal gait speed, WHO-5, and TMIG-IC

OR; odds ratio, CI; confidence interval

## Discussion

### Principal findings

In this study, we examined the predictive ability of the CompBased-CAT and the relationship between CompBased-CAT subtests and the risk of MCI after two years in community-dwelling older adults. CompBased-CAT accurately predicted MCI occurrence within two years. Although several PC-based cognitive function tests have been reported, most studies were cross-sectional, and participants attended memory clinics [18]. This study demonstrated that PC-based cognitive function tests can be used to assess future MCI risk in community-dwelling older adults. We believe that by predicting the risk at the MCI stage, proactive measures against dementia can be implemented effectively.

### Participant characteristics

The participants had mean years of education and MMSE and TMIG scores of 13.4 years, 29.0, and 12.5, respectively, which were slightly better than those reported in similar studies [19, 20]. This may be because this study was an invitational venue survey, which may have been more likely to include participants who were highly interested in health and could commute to the venue. The incidence of MCI in this study was 35.2 per 1000 person-years. A systematic review reported that the incidence of MCI in population- or community-based studies was between 8.5 and 76.8 per 1000 person-years [21]. The incidence of MCI in this study was within this range. Although the participants in this study had slightly better physical and psychosomatic functions, the incidence of MCI showed a general value, indicating that the study participants comprised a reasonable sample.

### Predictive ability of the CompBased-CAT

The CompBased-CAT had an AUC, sensitivity, and specificity of 0.79, 0.76, and 0.75, respectively. Previous studies that examined the ability of PC-based cognitive function tests to discriminate MCI reported higher accuracies, with AUCs, sensitivities, and specificities of 0.80–0.97, 0.80–0.96, and 0.73–0.94, respectively [18]. However, these results were obtained from cross-sectional studies and cannot be meaningfully compared with the results of this study. Shimada et al. [22] longitudinally examined the predictive ability of the National Center for Geriatrics and Gerontology-Functional Assessment Tool (NCGG-FAT), a PC-based cognitive function test, to predict the development of dementia and reported an AUC of 0.57–0.71. This study similarly examined the predictive ability of CompBased-CAT, a PC-based cognitive function test, for the future development of MCI and exhibited better predictive ability (AUC = 0.79) than in NCGG-FAT. This tool is significant because it targets MCI and is considered useful for preventing dementia in older people. Recently, a machine learning-based approach was used to predict cognitive impairment using multiple variables, with high predictive accuracy of AUC (0.92), sensitivity (0.97), and specificity (0.83) [23]. This approach is based on the prediction of 35 covariates and is not useful for screening risks in clinical practice because it requires multiple assessments. CompBased-CAT can be performed in 10–15 min and does not require specialized staff, making it suitable for actual clinical settings.

### Comparison in each subtest of the CompBased-CAT

The baseline scores of each subtest of the CompBased-CAT were significantly lower in the MCI group, indicating that those at risk for MCI show functional impairment in multiple domains of cognitive function from an early stage. Previous studies [24, 25] have reported that MCI in multiple domains, such as memory, verbal fluency, and figure recognition, was observable several years before the diagnosis of dementia, and similar results were obtained using CompBased-CAT. As early symptoms of cognitive impairment are observed in multiple domains of cognitive function, an accurate assessment of MCI across many domains may not be possible by testing only a small number of selected domains. This tool assesses cognitive function in multiple domains and is useful for detecting the early stages of MCI.

The logistic regression analysis showed that the total Z-score was significantly associated with the occurrence of MCI, even after controlling for all covariates, and that the total Z-score could predict the occurrence of MCI independent of demographic factors, physical and mental functioning, and IADL ability. In addition, for each subtest Z-score of the CompBased-CAT, a significant association was observed with the occurrence of MCI, even after adjusting for all covariates for digit span forward, digit span backward, memory recall of item names, recognition of figures, and the Stroop task. The association with MCI was stronger for the forward (odds ratio [OR] = 2.11, *P* <0.01) and backward digit spans (OR = 2.21, *P* <0.01). Because digit span forward and backward tasks require memorizing numbers and processing information simultaneously (i.e., working memory) [26], they may predict MCI more accurately than simple memory skills such as immediate memory. Working memory is impaired in the early stages of Alzheimer’s dementia [27], suggesting the need to evaluate complex abilities, such as working memory and simple memory.

### Limitations

This study had several limitations. First, we defined MCI using the MMSE-only scores. Although it is recommended that MCI be evaluated using the MoCA [11, 28], previous studies have shown that the discriminative ability (AUC) of the MoCA and MMSE for MCI were 0.88 and 0.78, respectively. This indicates that the MMSE is slightly inferior to the MoCA; however, the MMSE has a certain degree of accuracy [29]. Moreover, we did not perform a definitive diagnosis of MCI in this study. Therefore, healthy individuals may have been included among the patients with MCI. However, because the purpose of this study was screening and not to make a definitive diagnosis, we used a practical measure to evaluate MCI. The incidence of MCI in this study was 35.2 per 1000 person-years, which is a reasonable value compared to that in previous studies (8.5 and 76.8 per 1000 person-years) [21]. Therefore, the evaluation of MCI in this study appears to have sufficient validity. Second, this study was a venue-invitation survey, which may have introduced selection bias, and the participants had relatively high levels of education and mental and physical functioning. Therefore, the results of this study should be interpreted with caution. Further studies with a wider range of participants are warranted. Finally, the number of outcome events in this study was 32; the number of explanatory variables entered was nine; and the number of events per variable (EPV) was 3.56. In the logistic regression model, previous studies have highlighted that when the EPV is <10, the confidence interval of the regression coefficient becomes wide, affecting the reliability of the results [30]. However, the confidence intervals obtained in this study were sufficiently narrow. Simulations in previous studies have shown that the effect of a low EPV is even smaller when the independent variable is a continuous number [31]. Therefore, in this study, the effect of a low EPV was minimal, and the results obtained were considered reliable. Finally, the study did not include internal validation because of the small number of MCI cases, which may have resulted in overfitting. In the future, increasing the number of participants and validating the results using different datasets may be necessary.

## Conclusion

This study examined the ability of CompBased-CAT to predict the occurrence of MCI within two years. The results showed good accuracy, with an AUC, sensitivity, and specificity of 0.79, 0.76, and 0.75, respectively, indicating that CompBased-CAT can detect MCI, which is an early stage of dementia. We hope the CompBased-CAT will be used in future community health checkups and events for older adults.
